# Supplementary material for: Stable isotopes reveal winter feeding in different habitats in blue, fin and sei whales migrating through the Azores
Source: R Soc Open Sci. 2019 Aug 14;6(8):181800. doi: 10.1098/rsos.181800 (PMC6731742; doi:10.1098/rsos.181800)
Supplement: Supplemental Methods and Results [file rsos181800supp1.docx]

**Supplementary material from “Stable isotopes reveal winter feeding in different habitats in blue, fin and sei whales migrating through the Azores”**

**Methods**

***Potential range and seasonal movements of whale species***

The summer feeding range of North Atlantic blue whales extends from the Scotian Shelf to Davis Strait in the west, and from Denmark Strait to Svalbard in the east [1]. Fin whales co-occur with blue whales in most of this range but on the eastern Atlantic their feeding grounds extend further south to the British islands, Bay of Biscay and Iberian coast [2]. Additionally, there is evidence that part of the fin whale population wintering in the western Mediterranean migrates towards the Atlantic during the summer months [3]. Satellite telemetry studies indicate that blue and fin whales seen in the Azores migrate to central Atlantic waters, between Greenland and Iceland [4]. A recent photo-identification match between the Azores and the Gulf of St. Lawrence indicates that blue whales seen in the Azores may spend the previous summer on either side of the North Atlantic basin [5]. All sei whales instrumented with satellite tags in the Azores migrated to the Labrador Sea, off Canada [6,7]. Still, two individuals were apparently heading east of Greenland when their signals were lost, suggesting individuals may use feeding grounds on both sides of the Atlantic [7].

The wintering grounds of these species remain unknown. Fin whales were acoustically detected along the Mid-Atlantic Ridge (16°-50° N, 24°-50°W) from late autumn through early spring, with higher detection rates north of 32°N during winter months [8]. Whaling records and historic and contemporary winter sightings of sei whales along the West African coast (from Morocco down to Senegal) [9,10] and of blue whales between Cape Verde and Mauritania [10-12] suggest part of the population may winter off the Northwest Africa coast.

Based on the above, we considered that blue and sei whales seen in the Azores may summer in the Northeast Atlantic (NEA) and Northwest Atlantic (NWA), and spend the 3-4 months preceding sampling in lower-latitude pelagic waters (Azores (AZ), North Africa,-NAF) or closer to the Northwest African coast (NAF-UPW). In the case of fin whales, we also considered the Iberia (IB), Bay of Biscay (BB) and western Mediterranean (MED) as plausible winter-spring habitats.

**Table S1.** Mean and standard deviation (SD) δ^15^N and δ^13^C values of potential prey taxa from the literature used to estimate the isotopic composition of prey groups in different regions, by season. Only studies that accounted for lipid through chemical extraction or mathematical correction and provided data for both δ^15^N and δ^13^C values, along with a descriptor of the variance and number of samples analysed, were included. Regions are: AZ-Azores, IB-Iberia, BB-Bay of Biscay, NAF-North Africa, NAF-UPW-North Africa Upwelling, NEA-Northeast Atlantic, NWA-Northwest Atlantic, MED-Mediterranean.

| Region | Location | Prey group | Lowest taxonomic ID | Year | Season | δ15N | | δ13C | | Nº | References |
| --- | --- | --- | --- | --- | --- | --- | --- | --- | --- | --- | --- |
|  |  |  |  |  |  | Mean | SD | Mean | SD | samples |  |
| AZ | Azores | Copepods | Copepods | 2009 | Spring | 5.16 | 0.63 | -20.45 | 0.56 | 52 | 13 |
| AZ | Azores | Euphausiids | Euphausiidae | 2009 | Spring | 6.39 | 0.66 | -19.94 | 0.66 | 27 | 13 |
| BB | Bay of Biscay | Euphausiids | *Meganyctiphanes norvegica* | 2001-10 | Autumn | 8.30 | 0.20 | -19.80 | 0.20 | 15 | 14 |
| IB | Iberia | Euphausiids | *Meganyctiphanes norvegica* | 2001 | Spring | 6.35 | 0.56 | -20.93 | 0.31 | 6 | 15 |
| IB | Iberia | Euphausiids | *Meganyctiphanes norvegica* | 2001 | Autumn | 7.55 | 0.57 | -21.10 | 0.20 | 6 | 15 |
| NAF | Cape Verde | Copepods | *Undinula vulgaris* | 2012 | Autumn | 4.68 | 0.31 | -19.49 | 0.38 | 3 | 16 |
| NAF | Cape Verde | Euphausiids | Euphausiid | 2012 | Autumn | 3.04 | 0.32 | -18.60 | 0.16 | 3 | 16 |
| NAF | Guinea Dome | Copepods | *Undinula vulgaris* | 2012 | Autumn | 4.95 | 0.85 | -19.14 | 0.13 | 6 | 16 |
| NAF | Guinea Dome | Copepods | *Calanoides* sp. | 2015 | Spring | 8.28 | 1.10 | -19.37 | 0.80 | 13 | 17 |
| NAF | Guinea Dome | Euphausiids | Euphausiacea | 2015 | Spring | 7.79 | 1.06 | -19.70 | 0.40 | 11 | 17 |
| NAF | Guinea-Bissau - Liberia | Copepods | *Undinula vulgaris* | 2012 | Autumn | 6.07 | 0.15 | -19.97 | 0.06 | 3 | 18 |
| NAF | Guinea-Bissau - Liberia | Euphausiids | Euphausiid | 2012 | Autumn | 4.45 | 0.26 | -19.90 | 0.24 | 4 | 18 |
| NAF | Western Tropical Atlantic | Copepods | *Undinula vulgaris* | 2012 | Autumn | 4.46 | 1.75 | -19.47 | 0.90 | 23 | 16 |
| NAF | Western Tropical Atlantic | Copepods | *Calanoides* sp. | 2015 | Spring | 6.19 | 1.16 | -19.50 | 0.60 | 19 | 17 |
| NAF | Western Tropical Atlantic | Euphausiids | Euphausiid | 2012 | Autumn | 4.23 | 1.60 | -18.78 | 0.67 | 10 | 16 |
| NAF | Western Tropical Atlantic | Euphausiids | Euphausiacea | 2015 | Spring | 6.60 | 0.96 | -19.22 | 0.73 | 24 | 17 |
| NAF | Western Tropical Atlantic | Euphausiids | *Meganyctiphanes norvegica* | 2015 | Spring | 8.00 | 0.81 | -19.17 | 0.05 | 2 | 17 |
| NAF | Western Tropical Atlantic | Euphausiids | *Thysanoessa* sp. | 2015 | Spring | 8.15 |  | -20.54 |  | 1 | 17 |
| NAF-UPW | Saharan bank | Copepods | *Calanoides* sp. | 2015 | Spring | 5.57 | 1.15 | -17.93 | 1.35 | 7 | 17 |
| NAF-UPW | Saharan bank | Euphausiids | Euphausiid | 2012 | Autumn | 5.98 | 0.31 | -21.30 | 0.20 | 4 | 18 |
| NAF-UPW | Saharan bank | Euphausiids | Euphausiacea | 2015 | Spring | 6.25 | 1.58 | -18.16 | 1.53 | 14 | 17 |
| NAF-UPW | Senegal-Mauritania | Copepods | *Calanoides carinatus* | 2012 | Autumn | 6.80 | 0.91 | -17.70 | 0.45 | 8 | 18 |
| NAF-UPW | Senegal-Mauritania | Copepods | *Undinula vulgaris* | 2012 | Autumn | 4.62 | 0.52 | -19.06 | 0.41 | 19 | 16, 18 |
| NAF-UPW | Senegal-Mauritania | Copepods | *Calanoides* sp. | 2015 | Spring | 8.30 | 1.09 | -18.34 | 1.18 | 27 | 17 |
| NAF-UPW | Senegal-Mauritania | Euphausiids | Euphausiid | 2012 | Autumn | 6.14 | 1.59 | -19.16 | 0.49 | 10 | 16, 18 |
| NAF-UPW | Senegal-Mauritania | Euphausiids | Euphausiacea | 2015 | Spring | 8.63 | 1.16 | -18.51 | 1.71 | 19 | 17 |
| NEA | Iceland Sea | Euphausiids | *Meganyctiphanes norvegica* | 2007 | Summer | 7.50 | 0.52 | -20.40 | 0.35 | 3 | 19 |
| NEA | Iceland Sea | Euphausiids | *Thysanoessa inermis* | 2007 | Summer | 8.70 | 0.17 | -20.20 | 0.69 | 3 | 19 |
| NEA | Iceland Sea | Euphausiids | *Thysanoessa longicaudata* | 2007 | Summer | 9.00 | 0.17 | -22.10 | 0.17 | 3 | 19 |
| NEA | Spitzbergen | Copepods | *Calanus finmarchicus* | 2007 | Summer | 8.60 | 0.20 | -21.60 | 0.01 | 4 | 20 |
| NEA | Spitzbergen | Copepods | *Calanus hyperboreus* | 2007 | Summer | 6.70 | 1.00 | -22.20 | 0.26 | 4 | 21 |
| NEA | Spitzbergen | Euphausiids | Euphausiidae | 2007 | Summer | 8.30 | 0.10 | -20.00 | 0.11 | 4 | 21 |
| NEA | Svalbard | Copepods | *Calanus hyperboreus* | 2003 | Summer | 8.83 | 0.85 | -23.76 | 1.37 | 104 | 22 |
| NEA | Svalbard | Copepods | *Calanus glacialis* | 2003 | Summer | 8.40 | 1.31 | -22.58 | 1.46 | 146 | 22 |
| NEA | Svalbard | Copepods | *Calanus glacialis* | 2008 | Summer | 9.10 | 0.25 | -20.83 | 0.27 | 9 | 20 |
| NEA | Svalbard | Copepods | *Calanus finmarchicus* | 2008 | Summer | 7.90 | 0.10 | -21.20 | 0.10 | 3 | 20 |
| NEA | Svalbard | Copepods | *Calanus hyperboreus* | 2008 | Summer | 8.44 | 0.42 | -20.66 | 0.27 | 7 | 20 |
| NEA | Svalbard | Euphausiids | *Meganyctiphanes norvegica* | 2003 | Summer | 11.50 | 0.35 | -21.70 | 0.17 | 3 | 22 |
| NEA | Svalbard | Euphausiids | *Thysanoessa longicaudata* | 2003 | Summer | 10.30 | 0.51 | -21.70 | 0.51 | 26 | 22 |
| NEA | Svalbard | Euphausiids | *Thysanoessa inermis* | 2003 | Summer | 9.40 | 1.20 | -22.30 | 1.20 | 16 | 22 |
| NEA | Svalbard | Euphausiids | Euphausiidae | 2008 | Summer | 8.85 | 0.33 | -20.80 | 0.57 | 6 | 20 |
| NWA | Gulf St Lawrence | Copepods | *Calanus finmarchicus* | 2006 | Summer | 8.30 | 0.32 | -22.70 | 0.32 | 10 | 23 |
| NWA | Gulf St Lawrence | Copepods | *Calanus hyperboreus* | 2006 | Summer | 8.00 | 0.22 | -22.40 | 0.22 | 5 | 23 |
| NWA | Gulf St Lawrence | Euphausiids | *Meganyctiphanes norvegica* | 2006 | Summer | 9.20 | 0.49 | -21.10 | 0.24 | 6 | 23 |
| NWA | SW Greenland | Euphausiids | *Meganyctiphanes norvegica* | 2003 | Summer | 8.50 | 0.40 | -19.00 | 0.00 | 2 | 24 |
| NWA | Western Greenland | Copepods | *Calanus* spp. | 2010 | Summer | 8.39 | 1.27 | -20.97 | 0.84 | 38 | 25 |
| NWA | Western Greenland | Euphausiids | *Meganyctiphanes norvegica* | 2010 | Summer | 11.37 | 1.23 | -21.96 | 0.45 | 40 | 25 |
| NWA | Western Greenland | Euphausiids | *Thysanoessa inermis* | 2010 | Summer | 10.04 | 0.95 | -21.71 | 0.67 | 59 | 26 |
| NWA | Western Greenland | Euphausiids | *Thysanoessa longicaudata* | 2010 | Summer | 8.64 | 1.30 | -22.60 | 0.67 | 21 | 26 |
| NWA | Western Greenland | Euphausiids | *Thysanoessa raschii* | 2010 | Summer | 9.26 | 0.66 | -21.49 | 0.54 | 61 | 26 |
| MED | Balearic Sea | Euphausiids | *Meganyctiphanes norvegica* | 2009 | Winter | 6.91 | 0.74 | -19.68 | 0.33 | 5 | 27 |

**Table S2.** Estimates of the mean and standard deviation (SD) δ^15^N and δ^13^C values of regions used in Bayesian stable isotope mixing models run for blue, fin and sei whales. The model for sei whales included isotopic data from copepods belonging to family Calanidae; the models for blue and fin whales included isotopic data from euphausiids belonging to family Euphausiidae. Regions are: AZ-Azores, IB-Iberia, BB-Bay of Biscay, NAF-North Africa, NAF-UPW-North Africa Upwelling, NEA-Northeast Atlantic, NWA-Northwest Atlantic, MED-Mediterranean.

| Regions | Season | Years | δ^15^N | | δ^13^C | | N |
| --- | --- | --- | --- | --- | --- | --- | --- |
|  |  |  | Mean | SD | Mean | SD |  |
| **Blue whale model** | |  |  |  |  |  |  |
| NAF-UPW | Autumn | 2012 | 6.09 | 1.33 | -19.77 | 1.08 | 14 |
| NAF-UPW | Spring | 2015 | 7.73 | 1.69 | -18.76 | 1.02 | 33 |
| NAF | Autumn | 2012 | 4.07 | 1.31 | -19.01 | 0.73 | 17 |
| NAF | Spring | 2015 | 7.06 | 1.12 | -19.39 | 0.68 | 38 |
| AZ | Spring | 2009 | 6.39 | 0.66 | -19.94 | 0.66 | 27 |
| NEA | Summer | 2003, 2007, 2008 | 9.60 | 0.79 | -21.55 | 0.72 | 64 |
| NWA | Summer | 2003, 2006, 2010 | 9.78 | 0.96 | -21.79 | 0.36 | 189 |
|  |  |  |  |  |  |  |  |
| **Fin whale model** | |  |  |  |  |  |  |
| NAF-UPW | Autumn | 2012 | 6.09 | 1.33 | -19.77 | 1.08 | 14 |
| NAF-UPW | Spring | 2015 | 7.73 | 1.69 | -18.76 | 1.02 | 33 |
| NAF | Autumn | 2012 | 4.07 | 1.31 | -19.01 | 0.73 | 17 |
| NAF | Spring | 2015 | 7.06 | 1.12 | -19.39 | 0.68 | 38 |
| AZ | Spring | 2009 | 6.39 | 0.66 | -19.94 | 0.66 | 27 |
| NEA | Summer | 2003, 2007, 2008 | 9.60 | 0.79 | -21.55 | 0.72 | 64 |
| NWA | Summer | 2003, 2006, 2010 | 9.78 | 0.96 | -21.79 | 0.36 | 189 |
| MED | Winter | 2009 | 6.91 | 0.74 | -19.68 | 0.33 | 5 |
| IB | Spring | 2001 | 6.35 | 0.56 | -20.93 | 0.31 | 6 |
| IB | Autumn | 2001 | 7.55 | 0.57 | -21.10 | 0.20 | 6 |
| BB | Autumn | 2001-2010 | 8.3 | 0.20 | -19.80 | 0.20 | 15 |
|  |  |  |  |  |  |  |  |
| **Sei whale model** |  |  |  |  |  |  |  |
| NAF-UPW | Autumn | 2012 | 5.26 | 1.20 | -18.66 | 0.76 | 27 |
| NAF-UPW | Spring | 2015 | 7.71 | 1.56 | -18.35 | 1.09 | 34 |
| NAF | Autumn | 2012 | 4.70 | 1.52 | -19.46 | 0.76 | 40 |
| NAF | Spring | 2015 | 7.04 | 1.53 | -19.45 | 0.68 | 32 |
| AZ | Spring | 2009 | 5.16 | 0.63 | -20.45 | 0.56 | 52 |
| NEA | Summer | 2003, 2007, 2008, 2013 | 8.52 | 1.10 | -22.89 | 1.34 | 277 |
| NWA | Summer | 2006, 2010 | 8.36 | 1.07 | -22.04 | 0.85 | 91 |

**References**

1 Sears R, Perrin WF. 2018 Blue Whale: *Balaenoptera musculus*. In *Encyclopedia of Marine Mammals* 3^rd^ edition (eds. B Würsig, KM Kovacs, JGM Thewissen). *Academic Press* (doi:10.1016/C2015-0-00820-6)

2 Edwards EF, Hall C, Moore TJ, Sheredy C, Redfern JV. 2015 Global distribution of fin whales *Balaenoptera physalus* in the post-whaling era (1980–2012). *Mammal Rev.* **45**, 197–214. (doi:10.1111/mam.12048)

3 Geijer CKA, Notarbartolo di Sciara G, Panigada S. 2016 Mysticete migration revisited: Are Mediterranean fin whales an anomaly? *Mammal Rev.* **46**, 284-296. (doi:10.1111/mam.12069)

4 Silva MA, Prieto R, Jonsen I, Baumgartner MF, Santos RS. 2013 North Atlantic blue and fin whales suspend their spring migration to forage in middle latitudes: Building up energy reserves for the journey? *PLoS One*. **8**, e76507. (doi:10.1371/journal.pone.0076507)

5 Sears R, Vikingsson G, Santos R, Steiner L, Silva M, Ramp C. 2015 Comparison of northwest Atlantic (NWA) and northeast Atlantic (NEA) blue whale (*Balaenoptera musculus*) photo-identification catalogues. 21st Biennial Conference on the Biology of Marine Mammals. San Francisco CA.

6 Olsen E, Budgell P, Head E, Kleivane L, Nottestad L, Prieto R, Silva MA, Skov H, Vikingsson GA,Waring GT et al. 2009 First satellite-tracked long-distance movement of a sei whale (*Balaenoptera borealis*) in the North Atlantic. *Aquat Mamm*. **35**, 313-318. (doi:10.1578/AM.35.3.2009.31)

7 Prieto R, Silva MA,Waring GT, Gonçalves JMA. 2014 Sei whale movements and behaviour in the north Atlantic inferred from satellite telemetry. *Endang Species Res*. **26**, 103-113. (doi:10.3354/esr00630)

8 Nieukirk SL, Mellinger DK, Moore SE, Klinck K, Dziak RP, Goslin J. 2012 Sounds from airguns and fin whales recorded in the mid-Atlantic Ocean, 1999–2009. *J Acoust Soc Am*. **131**, 1102-1112. (doi:10.1121/1.3672648)

9 Prieto R, Janiger D, Silva MA, Waring GT, Gonçalves JM. 2012 The forgotten whale: a bibliometric analysis and literature review of the North Atlantic sei whale *Balaenoptera borealis*. *Mammal Rev.* **42**: 235-272. (doi:10.1111/j.1365-2907.2011.00195.x)

10 Baines M, Reichelt M. 2014 Upwellings, canyons and whales: An important winter habitat for balaenopterid whales off Mauritania, northwest Africa. *J Cetacean Res Manag*. **14**, 57-67.

11 Jonsgård A. 1966 The distribution of Balaenopteridae in the North Atlantic ocean. In Whales, dolphins and porpoises. (ed. KS Norris). Los Angeles, California: University of California Press.

12 Reeves RR, Smith TD, Josephson EA, Clapham PJ, Woolmer G. 2004 Historical observations of humpback and blue whales in the North Atlantic ocean: clues to migratory routes and possibly additional feeding grounds. *Mar Mamm Sci*. **20**, 774-786. (doi:10.1111/j.1748-7692.2004.tb01192.x)

13 Colaço A, Giacomello E, Porteiro F, Menezes GM. 2013 Trophodynamic studies on the Condor seamount (Azores, Portugal, North Atlantic). *Deep Sea Res II*. **98**, 178-189. (doi:10.1016/j.dsr2.2013.01.010)

14 Chouvelon T, Spitz J, Caurant F, Mèndez-Fernandez P, Chappuis A, Laugier F, Le Goff E, Bustamante P. 2012 Revisiting the use of δ^15^N in meso-scale studies of marine food webs by considering spatio-temporal variations in stable isotopic signatures – The case of an open ecosystem: The Bay of Biscay (North-East Atlantic). *Prog Oceanogr.* **101**, 92-105. (doi:10.1016/j.pocean.2012.01.004)

15 Bentaleb I, Martin C, Vrac M, Mate B, Mayzaud P, Siret D, de Stephanis R, Guinet C. 2011 Foraging ecology of Mediterranean fin whales in a changing environment elucidated by satellite tracking and baleen plate stable isotopes. *Mar Ecol Prog Ser*. **438**, 285-302. (doi: 10.3354/meps09269)

16 Sandel V, Kiko R, Brandt P, Dengler M, Stemmann L, Vandromme P, Sommer U, Hauss H. 2015 Nitrogen fuelling of the pelagic food web of the Tropical Atlantic. *PLoS ONE.* **10**, e0131258. (doi:10.1371/journal.pone.0131258)

17 Bode A, Hernandez-Leon S. 2018. Trophic diversity of plankton in epipelagic and mesopelagic layers of the tropical and equatorial Atlantic determined with stable isotopes. *Diversity*. **10,** 48. (doi:10.3390/d10020152)

18 Bode M, Hagen W, Schukat A, Teuber L, Fonseca-Batista D, Dehairs F, Auel H. 2015 Feeding strategies of tropical and subtropical calanoid copepods throughout the eastern Atlantic Ocean – latitudinal and bathymetric aspects. *Prog Oceanogr*. **138**, 268-282. (doi:10.1016/j.pocean.2015.10.002)

19 Petursdottir H, Gislason A. 2009 Trophic interactions and energy flow within the pelagic ecosystem in the Iceland Sea. *ICES CM* 2009/A:08

20 Hallanger IG, Ruus A, Warner NA, Herzke D, Evenset A, Schøyen M, Gabrielsen GW, Borgå K. 2011. Differences between Arctic and Atlantic fjord systems on bioaccumulation of persistent organic pollutants in zooplankton from Svalbard. *Sci Total Environ*. **15**, 2783-2795. (doi: 10.1016/j.scitotenv.2011.03.015)

21 Hallanger IG, Warner NA, Ruus A, Evenset A, Christensen G, Herzke D, Gabrielsen GW, Borgå K. 2011. Seasonality in contaminant accumulation in Arctic marine pelagic food webs using trophic magnification factor as a measure of bioaccumulation. *Environ Toxicol Chem*. **30**, 1026-1035. (doi: 10.1002/etc.488)

22 Søreide JE, Hop H, Carroll ML, Falk-Petersen S, Hegsethc EN. 2013 Seasonal food web structures and sympagic–pelagic coupling in the European Arctic revealed by stable isotopes and a two-source food web model. *Prog Oceanogr.* **71**, 59-87. (doi: 10.1016/j.pocean.2006.06.001)

23 Lavoie RA, Hebert CE, Rail JF, Braune BM, Yumvihoze E, Hill LG, Lean DRS. 2010 Trophic structure and mercury distribution in a Gulf of St. Lawrence (Canada) food web using stable isotope analysis*. Sci Total Environ.* **408**, 5529-5539. (doi: 10.1016/j.scitotenv.2010.07.053)

24 Møller P. 2006 Lipids and stable isotopes in marine food webs in West Greenland. Trophic relations and health implications. Phd thesis. National Environmental Research Institute, Denmark. (http://www2.dmu.dk/1_viden/2_Publikationer/3_Ovrige/rapporter/phd_PEM.pdf)

25 Bode A, Agersted MD, Nielsen TG, Basedow SL, Petursdottir H, Gislason A. 2014. Stable C and N isotopes and percent C as well as N composition of plankton from the North Atlantic. PANGAEA. (doi: 10.1594/PANGAEA.837299)

26 Valls MM. 2017 Trophic ecology in marine ecosystems from the Balearic Sea (Western Mediterranean). PhD thesis. Universitat de les Iles Balears. (https://www.tesisenred.net/handle/10803/461496)

27 Barría C, Navarro J, Coll M. 2017. Trophic habits of an abundant shark in the northwestern Mediterranean Sea using an isotopic non-lethal approach. *Estuar Coast Shelf Sci*. **207**, 383-390. (doi:1016/j.ecss.2017.08.021)

**Results**

**Table S3** Summary of results of one-way ANOVAs and Student’s *t*-test assessing differences in δ^15^N and δ^13^C values among whale species (blue, fin and sei whales) and among sexes, seasons and years for fin and sei whales. Only factors for which the PERMANOVA indicated a statistically significant effect on stable isotopes were tested with univariate analysis (except for the comparison between spring and summer isotope values for fin whales). Statistically significant (p<0.05) pairwise comparisons based on post hoc Tukey HSD test are shown in the table.

|  |  |  | δ^15^N value | | |  | δ^13^C value | | |
| --- | --- | --- | --- | --- | --- | --- | --- | --- | --- |
| Analyses | Factor | df | Student’s *t* or ANOVA *F* | *P* | Post hoc Tukey HSD |  | Student’s *t* or ANOVA *F* | *P* | Post hoc Tukey HSD |
| Inter-species |  | 2, 92 | *F*=6.21 | **0.003** | fin–sei |  | *F*=44.61 | **<0.001** | blue–fin, blue–sei, fin–sei |
| Fin whale | Sex | 40 | *t*=0.37 | 0.713 |  |  | *t*=1.60 | 0.118 |  |
|  | Season | 38 | *t*=-0.92 | 0.365 |  |  | *t*=0.81 | 0.423 |  |
|  | Year | 3, 34 | *F*=5.89 | **0.002** | 2008–2010, 2008–2013, 2008–2014 |  | *F*=27.25 | **<0.001** | 2008–2013, 2008–2014, 2010–2014 |
| Sei whale | Season | 2, 27 | *F*=0.32 | 0.731 |  |  | *F*=28.86 | **<0.001** | spring–summer, spring–autumn, summer–autumn |
|  | Year | 5, 27 | *F*=9.74 | **<0.001** | 2005–2014, 2008–2014, 2009–2014 |  | *F*=4.49 | **0.004** | 2008–2012, 2008–2014, 2009-2012 |

**
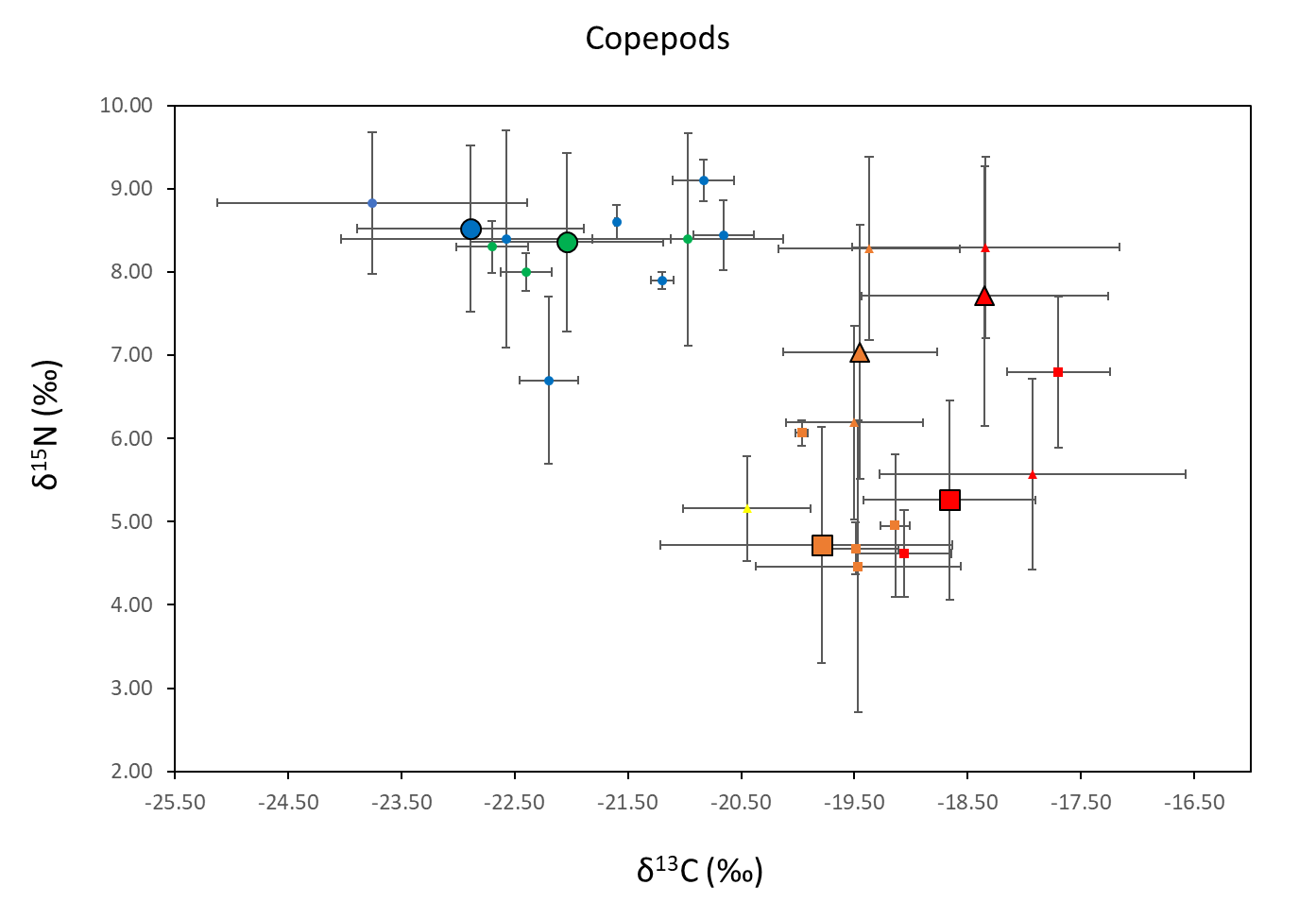
**

**
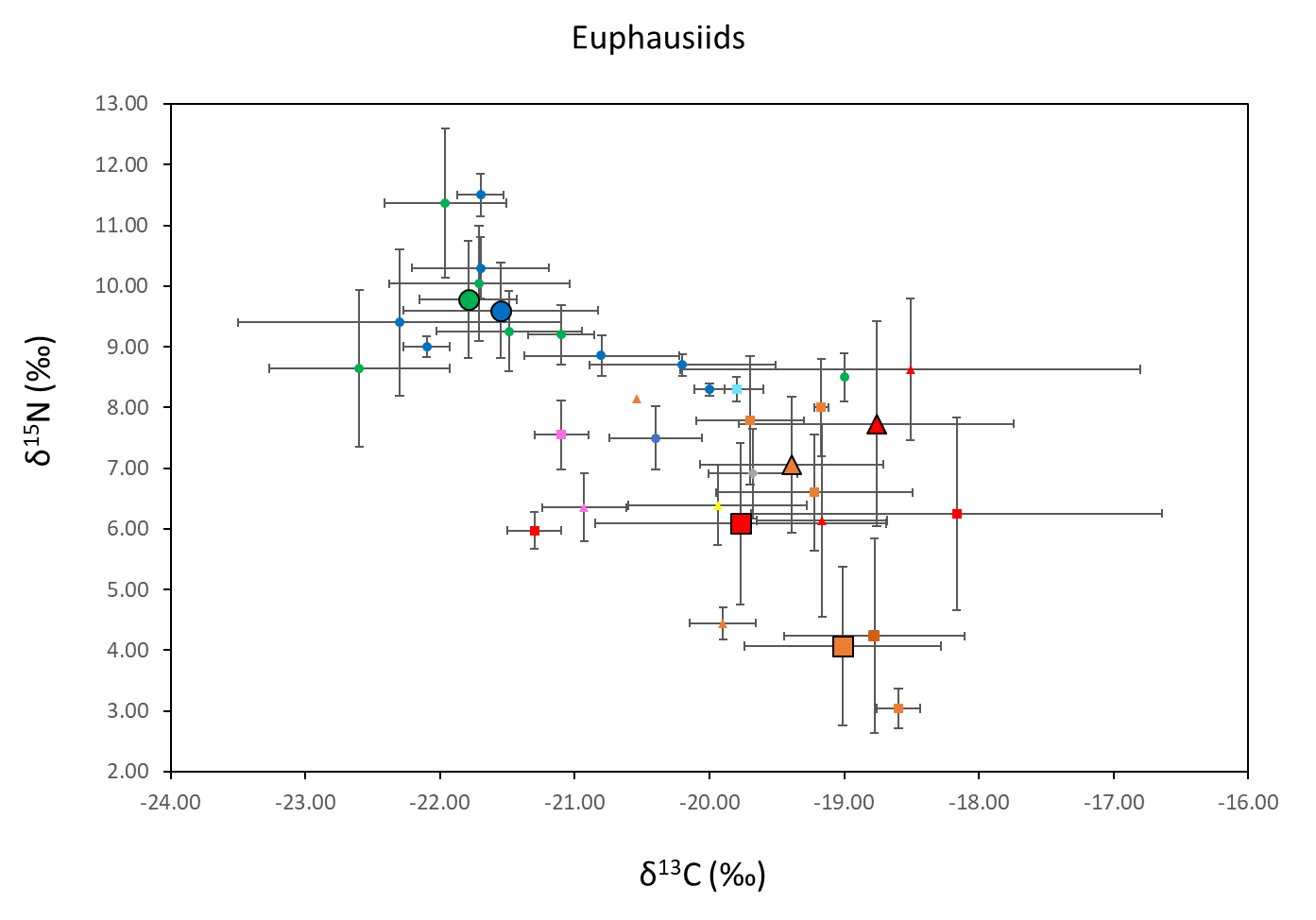
**

**Figure S1** - Mean and standard deviation (SD) δ^15^N and δ^13^C values of potential prey items from different regions and seasons. Copepods are shown in the top plot and euphausiids in the bottom plot. Regions are represented by different colours: AZ-Azores (yellow), IB-Iberia (pink), BB-Bay of Biscay (light blue), NAF-North Africa (orange), NAF-UPW-North Africa Upwelling (red), NEA-Northeast Atlantic (dark blue), NWA-Northwest Atlantic (green), MED-Mediterranean (gray). Seasons are represented by different symbols: Winter (diamond), Spring (triangle), Summer (circle), Autumn (square). Smaller symbols represent mean (SD) isotopic values of individual taxa and larger symbols represent the weighted average (SD) by region and season.

**Table S4 –** Percentage mean and SD contribution of different regional sources to the diet of blue, fin and sei whales estimated using Bayesian isotopic mixing models. Regions are: AZ-Azores, IB-Iberia, BB-Bay of Biscay, NAF-North Africa, NAF-UPW-North Africa Upwelling, NEA-Northeast Atlantic, NWA-Northwest Atlantic, MED-Mediterranean.

|  |  | Blue whale | | Fin whale | | | Sei whale | | | |
| --- | --- | --- | --- | --- | --- | --- | --- | --- | --- | --- |
|  |  |  |  |  | |  | Spring | | Summer | |
| Region-season | Season | Mean | SD | Mean | SD | | Mean | SD | Mean | SD |
| NAF-UPW-aut | Autumn | **20.0** | 16.7 | 4.5 | 4.5 | | **39.3** | 15.9 | **27.8** | 14.7 |
| NAF-UPW-spr | Spring | 7.5 | 5.6 | 2.7 | 2.1 | | **18.8** | 9.5 | **19.8** | 10.9 |
| NAF-aut | Autumn | **27.9** | 12.8 | 4.1 | 3.6 | | 13.5 | 10.7 | 13.1 | 8.9 |
| NAF-spr | Spring | 10.3 | 8.5 | 3.2 | 2.7 | | 11.7 | 9.1 | 12.6 | 8.8 |
| AZ | Spring | **18.1** | 16.6 | 4.3 | 4.2 | | 7.8 | 5.8 | 14.0 | 11.0 |
| NEA | Summer | 8.3 | 5.8 | 3.1 | 2.3 | | 4.2 | 2.6 | 5.9 | 4.3 |
| NWA | Summer | 8.0 | 5.7 | 3.1 | 2.4 | | 4.8 | 3.1 | 6.8 | 4.9 |
| MED | Winter |  |  | 3.6 | 3.2 | |  |  |  |  |
| IB-spr | Spring |  |  | **63.6** | 11.5 | |  |  |  |  |
| IB-aut | Autumn |  |  | 4.8 | 5.3 | |  |  |  |  |
| BB-aut | Autumn |  |  | 3.0 | 2.3 | |  |  |  |  |
